# Numerical Simulation of Gravitactic Bioconvection with Nanoparticles: An Application of Solids Removal in Wastewater Using a Thermal Source

**DOI:** 10.3390/mi16050553

**Published:** 2025-04-30

**Authors:** Alejandra M. Mil-Martínez, René O. Vargas, Aldo Gómez-López, Alejandro Zacarías, Juan P. Escandón, Enrique García-Leal, Rubén Mil-Martínez

**Affiliations:** 1 Escuela Militar de Ingeniería, Universidad del Ejército y la Fuerza Aérea, Av. Industria Militar No. 261, Col. Lomas de San Isidro, Naucalpan de Juárez 53960, Mexico; alemilm24@gmail.com; 2 Departamento de Termofluidos, SEPI-ESIME Azcapotzalco, Instituto Politécnico Nacional, Avenida de las Granjas No. 682, Colonia Santa Catarina, Alcaldía Azcapotzalco 02250, Mexico; azacarias@ipn.mx (A.Z.); jescandon@ipn.mx (J.P.E.); 3 Sección Mecánica, Departamento de Ingeniería, FES Cuautitlán, Universidad Nacional Autónoma de México, Av. Teoloyucan Km 2.5, Col. San Sebastián Xhala, Cuautitlán Izcalli 54714, Mexico; aldo.gl@comunidad.unam.mx; 4 Ingeniería Química, Facultad de Estudios Superiores Zaragoza, Universidad Nacional Autónoma de México, Batalla 5 de Mayo s/n esquina Fuerte de Loreto, Colonia Ejército de Oriente, Alcaldía Iztapalapa 09230, Mexico; egl_garcia@comunidad.unam.mx

**Keywords:** bioconvection, nanoparticles, *Paramecium caudatum*, thermophoresis, thermal source

## Abstract

The results of numerical simulations of gravitactic bioconvection influenced by nanoparticles suspended in water are analyzed. In this work, two cases are established which consider the removal of nanometric particles suspended in wastewater. The competence among the bioconvection of *Paramecium caudatum*, natural convection and buoyancy of nanoparticles phenomena in an aqueous suspension is presented. The position of a thermal source to control the orientation of microorganisms when swimming is analyzed. Numerical simulations are carried out using the finite difference method in an ADI scheme, employing stream-vorticity formulations and equations for microorganisms, nanoparticle concentration, and energy. The percentage of nanoparticles is considered using the Rayleigh number, which includes the effect of Brownian and thermophoretic parameters. At low values of the Brownian parameter $\delta_{Bm}=0.1$, thermophoretic parameter $\delta_{Tm}=0.1$, and the nanoparticles Rayleigh number $0.005<Ra_{n}<0.015$, the swimming of microorganisms contributed to streamlines across which nanoparticles traveled in response to a thermal control source. Thus, the results obtained suggest an alternative approach to the removal of solids such as heavy metals in polluting waters. The development of this type of technology will help in the bioremediation of wastewater.

## 1. Introduction

Today, bioconvection is widely studied due to the convective instability caused by swimming microorganisms. It is essential to analyze the main parameters that can provide information to implement this phenomenon in applications such as bioreactor optimization, the pharmaceutical industry, biodiesel use, and water treatment plants. Microorganisms swimming with nanoparticles saturated in layers of porous medium were analytically studied using linear stability by Nield and Kuznetsov [1]. They reported a second-order effect on the energy equation and a contribution of the nanoparticles to the buoyancy effect. In addition, they found that the basic nanoparticle distribution influences the critical thermal Rayleigh number. Moreover, Kuznetsov [2] analyzed the onset of bioconvection due to gyrotactic microorganisms that perturbed the system in a horizontal layer filled with nanofluids. Shivani and Sharma [3] performed numerical simulations of a fluid saturated by a porous medium, analyzing thermo-bioconvection combined with the perturbing effect of vertical flow on the bioconvection pattern. Ankita and Sanjalee [4] worked with a nanofluid layer subject to horizontal parallel walls. They found that decreasing the cell diffusivity stabilized the system, while increasing the Rayleigh and Peclet numbers resulted in perturbation. Akbar et al. [5] presented numerical calculations for a thermodynamic analysis of bioconvection in peristaltic flow with nanofluids and gyrotactic microorganisms. Their analysis included Arrhenius activation and found that the temperature increased when the activation parameters were higher. Furthermore, they suggested that heat sources and the thermophoresis parameters improved the heat transfer rate in the walls. They minimized the entropy by increasing the porosity parameter. Bioconvection modified by nanofluid flow in a crown cavity was investigated by Umair [6], considering how the density of gyrotactic microorganisms intensified the bioconvection parameters. Mohammadreza et al. [7] conducted research on the natural convection of nanofluids with oxytactic microorganisms in a square cavity in porous media, investigating how microorganisms modified the heat transfer rate. Li et al. [8] performed a numerical analysis of the impact of melting transport influenced by magnetohydrodynamic nanofluid microorganisms. Naveed Khan et al. [9] analyzed the swirling flow of a Maxwell fluid in the boundary layer in the bioconvection phenomenon of chemotaxis. They reported that the concentration gradient intensifies the thermal profile, while mass transportation improves with the thermal gradient. Mezentseva and Smorodin [10] reported a study of gravitactic bioconvection in a horizontal layer considering a nonisothermal liquid with a free, nondeformable boundary. They showed that there is no relationship between the long-wave concentration Rayleigh number and the thermal Rayleigh number.

Different studies of bioconvection obtained through numerical simulations have been reported, including study of the magnetohydrodynamic bionanoconvective flow of a micropolar fluid [11], the bioconvection of gyrotactic microorganisms under a non-uniform magnetic field [12], the numerical simulation of the nanoflow of a Powell–Eyring fluid through the Riga surface [13], which are interesting works on bioconvection. Many other studies describe the effects of the main bioconvective parameters under different flow and boundary conditions using stability analysis or numerical simulation [14,15,16,17,18,19,20,21]. The works previously cited reflect the efforts that have been made to understand the hydrodynamics and fundamental parameters involved, such as the Rayleigh, Peclect, and Smith numbers, to mention a few. That is why this phenomenon can now be used in different fields, such as in the pharmaceutical industry, agriculture, and even water remediation. The pollution of different ecosystems is caused by urbanization, current agricultural methods, and rapid industrialization. Industrial pollution has affected the air, soil, and water we use [22,23]. Specifically, the repercussions for health caused by heavy metals in humans and animals have highlighted the critical importance of finding an alternative solution to reduce their effect on ecosystems [24]. Various efforts have been made to reduce contaminants in water through the use of microorganisms, that is, by the bioremediation of water. Katarzyna and Christel [25] studied a variety of mechanisms responsible for the adaptation of microorganisms to high concentrations of heavy metals. They reported the capacity of microorganisms for metal accumulation that could be exploited to remove, concentrate, and recover metals from polluted sites. They highlighted that this biotechnological solution is an environmentally friendly, inexpensive, and efficient means of environmental restoration. Abatenh et al. [26] reported that the nutritional capacity of microorganisms varies widely. Bioremediation can be used in the degradation, eradication, immobilization, or detoxification of various chemical wastes and physical hazardous materials through the action of microorganisms. Furthermore, microbial strains have plant-growth promoting and contaminant-degrading abilities and may offer an efficient, cost-effective, and environmentally safe option for bioremediation and phytoremediation [27]. Herman et al. [28] worked with cultures of the aquatic unicellular organism *Paramecium caudatum*. Their results showed that paramecia could conveniently be used as a model bioindicator system. Zahra et al. [29] presented a recent research work which focused on the molecular identification of metal-resistant *Paramecium* species and their ability to take up different metals, such as Cd^2+^, Cu^2+^, Zn^2+^, and Pb^2+^. Some experimental studies on *Paramecium caudatum* and the effect of temperature were performed in 1972 and 1977 by K. Tawada and F. Oosawa [30], and Yasuo Nakaoka and Fumio Oosawa [31], respectively. In both works, the authors reported that the swimming speed of *Paramecium caudatum* was altered in relation to the temperature gradient. Unsurprisingly, although these microorganisms swim against gravity (gravitaxis), they also exhibited thermotaxis because the thermal gradient increased their swimming speed, and microorganisms were accumulated at optimal temperatures. Our research group first presented the stability and numerical simulation of a gravitactic microorganism, *Paramecium caudatum* [15]. Secondly, the dynamics of the bioconvection process of gravitactic microorganisms was studied considering how discrete thermal sources enhance the spatial orientation and distribution of the microorganisms [32]. In this work, the control of the swimming behavior of microorganisms using thermal sources was analyzed numerically. The study focused on the removal of nanoparticles that were spatially distributed and considered heavy metals when added to a water suspension in a rectangular enclosure. The results were obtained through numerical simulations. In the literature, similar work, where microorganisms are controlled with thermal sources to remove nanoparticles that emulate heavy metals as contaminants in water, has not been reported with respect to implementation as a novel solution for solids removal in wastewater.

## 2. Problem Formulation

**Figure 1:**
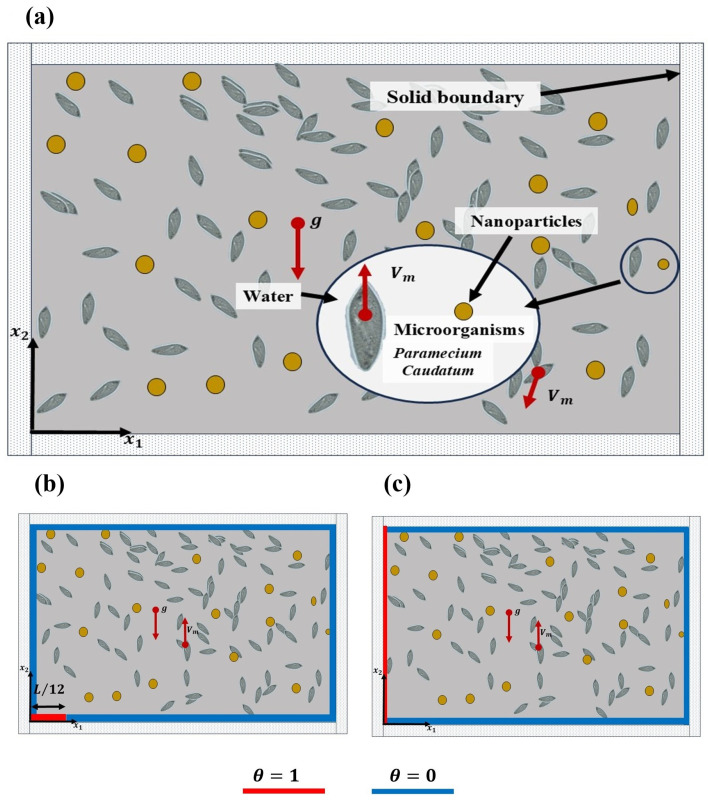
(**a**) Description of the bioconvective process with the presence of nanoparticles (heavy metals), (**b**) heat source in a bottom wall section, and (**c**) heat source in the side wall.

This work considers a rectangular enclosure containing nanoparticles with a suspension of *Paramecium caudatum* as gravitactic microorganisms. An initial condition with respect to the concentration of microorganisms and nanoparticles to obtain a uniform spatial distribution was proposed by [1,15] (see Equations (13) and (14), respectively). The thermal sources are configured here to control the microorganisms and prevent concentration of nanoparticles at the bottom and lateral boundaries of the wall. A two-dimensional rectangular cavity of height *H* and width *L* is established using the Cartesian coordinate system ($x_{1}$,$x_{2}$). In this case, the microorganisms have a uniform distribution and tend to swim vertically upward $\left(x_{2}\right)$ (see Figure 1a). In this study, the particles are considered to be dispersed with no sedimentation. It is assumed to be a perturbed state in which the particles are diffused. We focus on the controlled swimming of microorganisms that remove nanoparticles and then consider thermotaxis. A constant dimensionless wall temperature θ=0 is set and for the thermal sources θ=1. Two cases are analyzed to demonstrate the use of thermal sources as a means of controlling the swimming of the microorganisms in two different zones of the cavity. In the first case, the source is located in the bottom-left corner (see Figure 1b). In the second case, the source is placed on the left wall (see Figure 1c). This thermal source configuration is selected based on previous work [32], where the influence of the thermal source on the swimming behavior of microorganisms is presented. It is well known that at a value of 1708 for the Rayleigh number, natural convection can be observed. In [15,32], it is suggested that at a bioconvective Rayleigh number Ra = 1900, a plume shape concentration occurs, with two or three rolls present. This suggests that the system in this range is stable when bioconvection dominates. Hence, these values are used in order to determine the relative effects of natural convection, bioconvection, and the buoyancy effect of nanoparticles. In relation to the thermal source, the thermophoretic and Brownian parameters are examined. The Schmidt, Peclect, and Lewis numbers are fixed to 1 to stabilize the system [3,4,7,32]. The Peclet number of nanoparticles ($Pe_{n}$) represents the ratio of nanoparticles to the diffusion of microorganisms. $Pe_{n}=0.1$ is used to ensure that the diffusion of microorganisms is greater than the velocity of the nanoparticles, promoting non-sedimentation. The effect of the Brownian and thermophoretic parameters on the swimming behavior of the microorganisms and their spatial distribution is analyzed.

### 2.1. Dimensionless Scales

The dimensionless variables are defined as follows:

(1) $$t=t^{\prime }\frac{D_{m}}{H^{2}},\;x_{1}=\frac{x_{1}^{\prime }}{H},\;x_{2}=\frac{x_{2}^{\prime }}{H},\;$$

(2) $$u=u^{\prime }\frac{H}{D_{m}},\;v=v^{\prime }\frac{H}{D_{m}},\;\omega =\omega^{\prime }\frac{H^{2}}{D_{m}},\;$$

(3) $$\Psi =\frac{\Psi^{\prime }}{D_{m}},\;n=\frac{n^{\prime }}{\bar{n}},\;\bar{N}=\frac{\bar{n}-n_{b}}{n_{u}-n_{b}}\;\theta =\frac{T-T_{h}}{T_{h}-T_{c}}\;,$$

(4) $$\phi =\frac{\phi -\phi_{0}}{\phi_{0}}\;,$$

where $x_{1}^{\prime }$ and $x_{2}^{\prime }$ denote the coordinate system, *H* is the cavity height, $D_{m}$ is the diffusion coefficient of the microorganisms, $u^{\prime }$ and $v^{\prime }$ are components of the horizontal and vertical velocity, $T_{c}$ and $T_{h}$ are the cold and hot wall temperatures, $n_{u}$ and $n_{b}$ are the concentrations of microorganisms at the top and bottom, $\Psi^{\prime }$ is the stream function, and ϕ is the nanoparticles volume fraction.

### 2.2. Mathematical Model

For this system, the bioconvection of gravitactic microorganisms is governed by the set of Equations (5)–(9), adapted from other works [1,2,3,4,15]. Therefore, the dimensionless governing equations are as follows:

(5) $$\frac{\partial^{2}\Psi }{\partial x_{i}^{2}}=-\omega ,$$

(6) $$\frac{\partial \omega }{\partial t}+u_{i}\frac{\partial \omega }{\partial x_{i}}=Sc\frac{\partial^{2}\omega }{\partial x_{i}^{2}}-ScRa\frac{\partial n}{\partial x_{1}}+LeScRa_{T}\frac{\partial \theta }{\partial x_{1}}-LeScRa_{n}\frac{\partial \phi }{\partial x_{1}},$$

where $u_{j}$, Ra, Sc, *n* are the velocity vector, the Rayleigh bioconvection number, the Schmidt bioconvection number, and the concentration of microorganisms, respectively. Le, $Ra_{T}$, $Ra_{n}$ are the Lewis number, the thermal Rayleigh number, and the nanoparticle Rayleigh number, respectively.

(7) $$\frac{\partial n}{\partial t}=-\frac{\partial (u_{i}+Pe\gamma_{2})n}{\partial x_{i}}+\frac{\partial^{2}n}{\partial x_{i}^{2}},$$

(8) $$\frac{\partial \theta }{\partial t}+u_{i}\frac{\partial \theta }{\partial x_{i}}=Le\frac{\partial^{2}\theta }{\partial x_{i}^{2}}+r_{\rho }\delta_{Bm}\frac{\partial \phi }{\partial x_{i}}\frac{\partial \theta }{\partial x_{i}}+r_{\rho }\delta_{Tm}{\left(\frac{\partial \theta }{\partial x_{i}}\right)}^{2},$$

(9) $$\frac{\partial \phi }{\partial t}+u_{i}\frac{\partial \phi }{\partial x_{i}}=\delta_{Bm}\frac{\partial^{2}\phi }{\partial x_{i}^{2}}+\delta_{Tm}\frac{\partial^{2}\theta }{\partial x_{i}^{2}}.$$

where Pe and $\gamma_{2}$ are the bioconvection Peclet number and a vertical unit vector, respectively. $r_{\rho }$, $\delta_{Bm}$, $\delta_{Tm}$, are the ratio density, Brownian diffusion parameter, and thermophoretic parameter, respectively The dimensionless numbers are defined as follows:

(10) $$\begin{array}{cc} Ra & =g\bar{n}\vartheta \Delta \rho H^{3}/\rho_{w}\nu D_{m},\\ Ra_{T} & =g\beta \left(T_{h}-T_{c}\right)H^{3}/\nu \alpha ,\\ Ra_{n} & =g\left(\rho_{p}-\rho_{f}\right)\phi_{0}H^{3}/\rho_{w}\nu \alpha ,\\ Sc & =\nu /D_{m},\\ Pe & ={\vec{V}}_{m}H/D_{m},\\ Pe_{n} & ={\vec{W}}_{0}H/D_{m},\\ Le & =\alpha /D_{m},\\ \delta_{Tm} & =D_{T}\left(T_{h}-T_{c}\right)/D_{m}T_{C}\phi_{0},\\ \delta_{Bm} & =D_{B}/D_{m},\\ r_{\rho } & =\rho_{p}c_{p}\phi_{0}/\rho_{f}c_{f}, \end{array}$$

where *g*, $\bar{n}$, ϑ, Δρ, $\left(\Delta \rho =\rho_{m}-\rho_{w}\right)$, ν, and ${\bar{V}}_{m}$ are the gravity, the dimensional average microorganisms’ concentration, the microorganisms volume, the difference between the microorganism and the water density, the kinematic diffusivity, and the upward microorganisms’ velocity, respectively.

### 2.3. Boundary Conditions

The set of Equations (5)–(9) is subjected to the following boundary conditions: rigid on the top and bottom walls and non-slip on the side walls, since the water suspension is an incompressible fluid and the characteristic length scale is above 300 nm [33]. Furthermore, there is no flux of microorganisms through the walls.

(11) $$\Psi =\frac{\partial \Psi }{\partial x_{1}}=0,\;\frac{\partial n}{\partial x_{1}}=0,\;\frac{\partial \phi }{\partial x_{1}}=0\;\;at\;x_{1}=0,A,$$

(12) $$\Psi =\frac{\partial \Psi }{\partial x_{2}}=0,\;nPe+nPe_{n}-\frac{\partial n}{\partial x_{2}}=0,\;\delta_{Bm}\frac{\partial \phi }{\partial x_{2}}+\delta_{Tm}\frac{\partial \theta }{\partial x_{2}}-Pe_{n}\phi =0\;at\;x_{2}=0,1,\;$$

where A=L/H=5, and $Pe_{n}$ are the cavity aspect ratio and the nanoparticle Peclet number.

### 2.4. Initial Conditions for Microorganisms, Temperature, and Nanoparticles Concentrations

To predict and analyze the diffusive state of bioconvection and nanoparticles under initial conditions, the basic state of the microorganims $n_{b}$ and nanoparticles $\phi_{b}$ is considered for the numerical simulations [1,15]:

(13) $$n_{b}=exp\left(Pex_{2}\right)\bar{N}Pe/exp\left(Pe\right)-1$$

(14) $$\phi_{b}=-\delta_{Bm}\theta_{b}+\left(1-\delta_{Bm}\right)x_{2}+\delta_{Bm}$$

where $\bar{N}$ is the average concentration and $\theta_{b}=0$ is the temperature basic state.

## 3. Numerical Simulations

**Figure 2:**
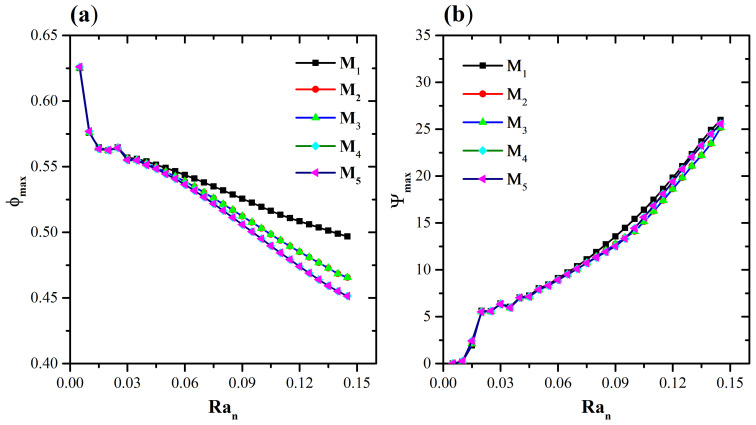
Mesh independence test for (**a**) $\phi_{max}$ versus $Ra_{n}$ and (**b**) $\Psi_{max}$ versus $Ra_{n}$ when $\delta_{Bm}=1.6$, $\delta_{Tm}=0.1$.

A cavity with a suspension of *Paramecium caudatum* as gravitactic and thermotactic microorganisms is considered. At time t = 0, the nanoparticles are evenly distributed in the cavity. To solve Equations (5)–(9), numerical simulations were performed using an Alternating Direction Implicit (ADI) method in finite differences, programmed in Fortran. When $f_{i,j}^{m+1}\leq \epsilon$, convergence is achieved; where *f* corresponds to the variables (ω,ψ,n,θ,ϕ), *m* is the iteration number, $\epsilon =1\times 10^{-6}$ is the value of the convergence criteria, and i,j are the points on the grid. To enhance simulation accuracy, an independence mesh analysis is developed. For the mesh refinement study, five uniform meshes are considered ($M_{1}-M_{5}$) (see Table 1). To test the appropriate mesh, we begin with $M_{1}$, then from ($M_{2}-M_{5}$). First, we refine it in the $x_{1}$-direction to choose a mesh that does not present significant variations in the solution. Secondly, in Figure 2a,b, the behavior of $\psi_{max}$ and $\phi_{max}$ is considered when $Ra_{n}$ increases, while $\delta_{Bm}=1.6$ and $\delta_{Tm}=0.1$ remain constant. A slight variation in $\psi_{max}$ and $\phi_{max}$ is observed, specifically, close to $Ra_{n}=0.14$. However, the values are almost identical throughout the resolution. Finally, in this work, $M_{3}$ is the mesh used for the numerical simulation, as it offers shorter computation times than $M_{4}-M_{5}$, while maintaining consistency of behavior across all resolutions and convergence stability.

## 4. Results and Discussion

The numerical results are obtained considering $Ra=1900,Ra_{T}=1708$, $Sc=Le=Pe=1,Pe_{n}=0.1$. $\delta_{Bm}=\left[0.1-1.9\right],\delta_{Tm}=\left[0.01-0.1\right],r_{\rho }=2.5$. The goal of establishing these values is to analyze reported cases [15] in which the effect of bioconvection and natural convection on the spatial distribution are determined at these values. In addition, the concentration gradient of the nanoparticles produces a destabilizing effect in the aqueous suspension, which has an effect on the energy and momentum equation. The values for $\delta_{Tm}$ and $\delta_{Bm}$ are varied by observing the competition of thermophoresis and Brownian diffusion with respect to the diffusion of microorganisms (see Figure 4, 5 and 9). The Rayleigh number of the nanoparticles $Ra_{n}$ is systematically increased from 0.05 to 0.14. The behavior at maximum values of nanoparticle concentration and streamlines is presented for different $Ra_{n}$. To understand the best configurations for removing solid nanoparticles in the water suspension, the microorganism *n* and nanoparticles ϕ concentrations, temperature θ, and streamlines Ψ contours are shown in this section.

### 4.1. First Case, Thermal Source at Bottom Wall Side

**Figure 3:**
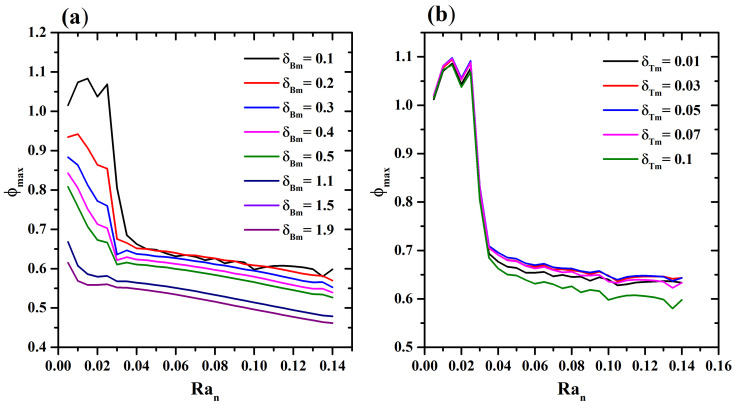
(**a**) Maximun value of nanoparticles concentrations $\phi_{max}$ as a function of $Ra_{n}$ with $\delta_{Tm}=0.1$ for different values of $\delta_{Bm}$, and (**b**) $\phi_{max}$ versus $Ra_{n}$ with $\delta_{Bm}=0.1$ for different values of $\delta_{Tm}$.

The spatial distribution when $\delta_{Bm}$ and $\delta_{Tm}$ are varied when a thermal source is used as a controller of the swimming behavior of the microorganisms is evaluated. The thermal source is located in the left corner of the bottom wall. Figure 3a shows the behavior of $\phi_{max}$ when $Ra_{n}$ increases for different values of $\delta_{Bm}$. An exponential decrease is shown for $\delta_{Bm}=0.1$. While for $\delta_{Bm}>0.2$ and $Ra_{n}>0.2$, a linear decrease is observed. When $\delta_{Tm}=0.1,\delta_{Bm}=\left[0.01-0.1\right]$, an exponential decrease is observed, as shown in Figure 3b, due to diffusion of the microorganisms.

**Figure 4:**
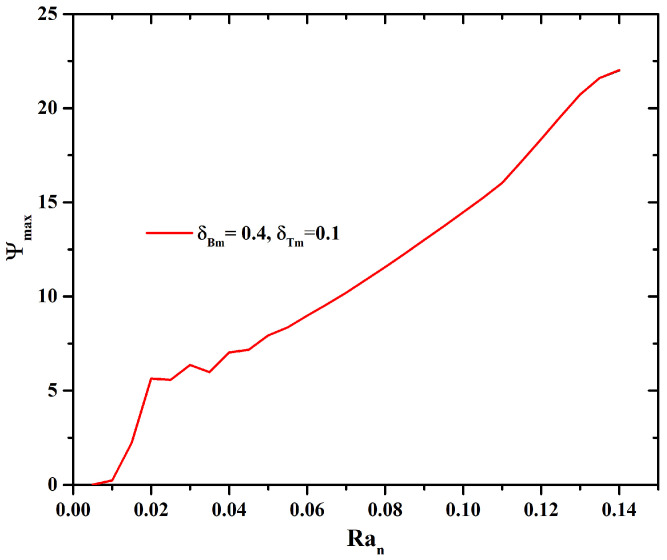
Streamline maximum value $\Psi_{max}$ as a function of $Ra_{n}$ with $\delta_{Bm}=0.4$ and $\delta_{Tm}=0.1$.

**Figure 5:**
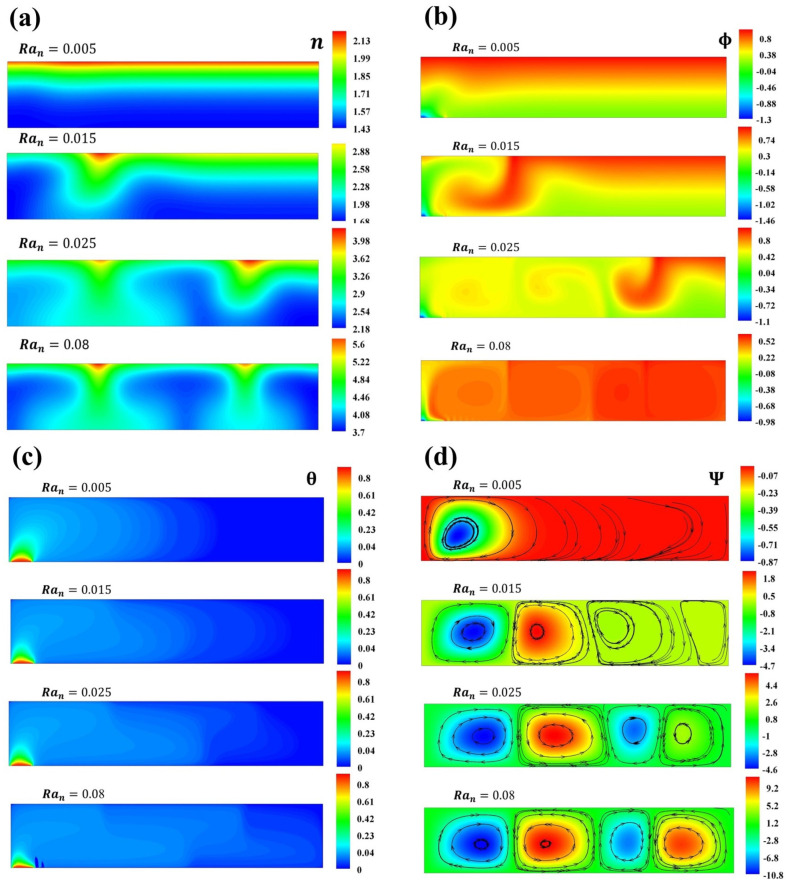
Concentration of (**a**) microorganisms, (**b**) nanoparticles, (**c**) temperature contours, and (**d**) streamlines for $Ra_{n}=0.005,0.015,0.025,0.08$.

No changes are observed when $\delta_{Bm}$ and $\delta_{Tm}$ are varied. Figure 4 presents the profile of Ψ when $Ra_{n}$ is varied. For $Ra_{n}=\left[0.015-0.05\right]$, subcritical values that cause instabilities are observed. The diffusion of heat due to the constant flow of heat increases the natural convection when $Ra_{n}$ increases, and the streamlines present higher values. The microorganism concentrations reflect the main driving force. Small perturbations in the nanoparticle concentration directly impact the velocity of the fluid. Figure 5 supports the results of Figure 3 and Figure 4 for $\delta_{Bm}=\delta_{Tm}=0.1$, with respect to the influence of the swimming thermal orientations on the spatial nanoparticle distributions. The pattern exhibited is that, with small fluctuations in nanoparticle concentrations, there is a preferred route for the microorganisms, resulting in microorganism and nanoparticle plumes with zones of higher concentrations. From one roll to two bioconvective cells are obtained.

**Figure 6:**
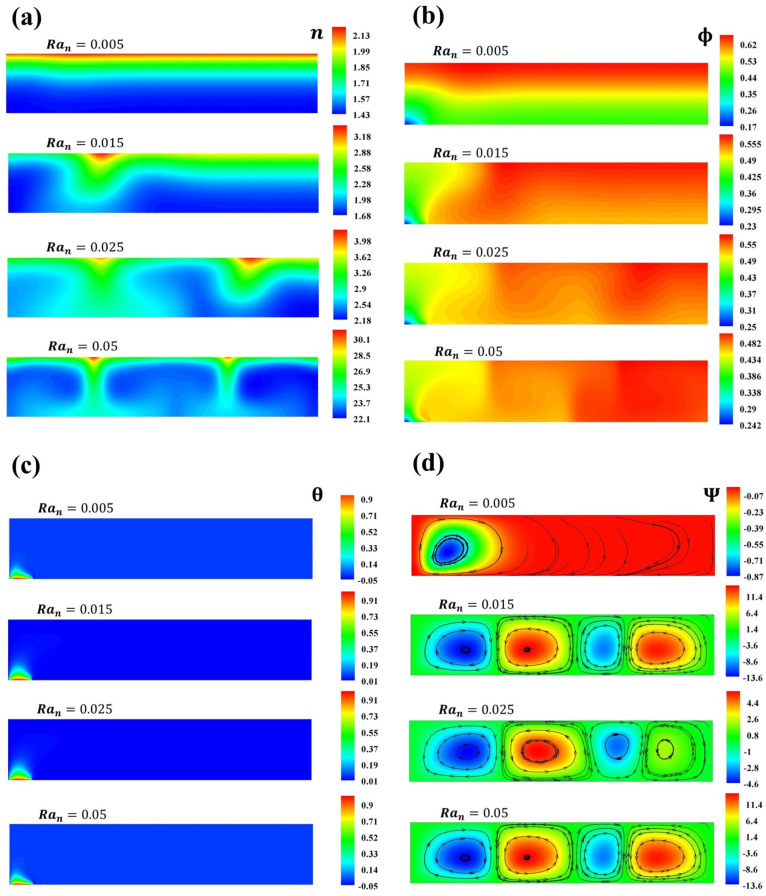
Concentration of (**a**) microorganisms and (**b**) nanoparticles, (**c**) temperature contours, and (**d**) streamlines for $Ra_{n}=0.005,0.015,0.025,0.05$.

**Figure 7:**
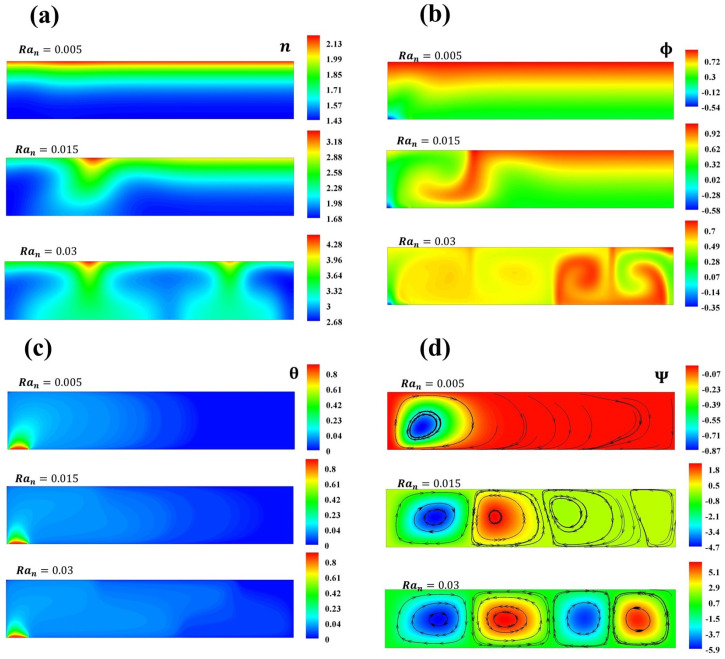
Concentration of (**a**) microorganisms and (**b**) nanoparticles, (**c**) temperature contours, and (**d**) streamlines for $Ra_{n}=0.005,0.015,0.03$.

Figure 6 illustrates the variation in n,Ψ,Φ, and θ when $\delta_{Bm}=1.1,\delta_{Tm}=0.1$ for different values of $Ra_{n}$. A comparison between the Brownian and thermophoretic effects stimulated by the thermal gradient is presented. The swimming of microorganisms drives the formation of concentration plumes of microorganisms and nanoparticles. It is worth noting that the Brownian and thermophoresis effects impact the streamlines, incrementing their values due to the thermal gradient. In contrast with Figure 7, when $\delta_{Bm}=0.1,\delta_{Tm}=0.05$, small perturbations of the thermophoretic parameter result in higher values on $\phi_{max}$ that are opposite to the thermal source, indicating that the thermophoresis effect dominates because of the thermal gradient. When $\delta_{Tm}<0.1$, the concentration of nanoparticles exhibits two plumes, one located in a higher concentration region; moreover, two plumes of microorganism concentrations and thermal diffusion are observed. As mentioned, the heat source orients the swimming of microorganisms, and these, in turn, orient to the nanoparticles in concentration plumes. For lower values of the thermophoretic parameter, a higher concentration of particles is observed away from the heat source, while higher values show a higher dispersion of particles [3,34].

### 4.2. Second Case, Thermal Source at Left Wall Side

The first case presented above in Figure 1b allowed us to observe the effect of the Brownian and thermophoretic parameters when nanoparticles with a higher density than that of water are suspended in water with microorganisms. The effects of the phenomena of natural convection, bioconvection, and the flotation effect caused by the presence of nanoparticles is presented. In this case, it was simulated that the bioconvective phenomenon dominates, while the other two cause perturbing effects. We observe that it is possible to use this configuration to remove nanoparticles when they are suspended in water with microorganisms.

In the following case study, natural convection did not have much impact due to the source configuration (see Figure 1c). Using this approach, the Brownian and thermophoretic effects on the bioconvective process are directly analyzed.

**Figure 8:**
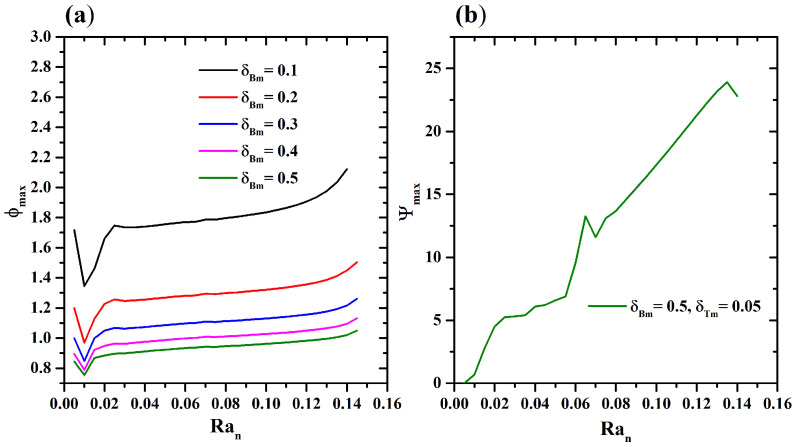
(**a**) Maximum value of nanoparticles concentrations $\phi_{max}$ as a function of $Ra_{n}$ with $\delta_{Tm}=0.05$ for different values of $\delta_{Bm}$, and (**b**) streamlines maximum value $\Psi_{max}$ versus $Ra_{n}$ with $\delta_{Tm}=0.05$ and $\delta_{Bm}=0.5$.

Figure 8a shows the profile of $\phi_{max}$ when $Ra_{n}$ is modified at different values of $\delta_{Bm}$, while $\delta_{Tm}=0.05$. A significant growth is shown, with a critical value of $Ra_{n}=0.01=Ra_{n_{cr}}$. After this value, the suspension is destabilized and higher values of Ψ are presented (see Figure 8b). No changes were observed at different values of $\delta_{Tm}$, which retained the same profile as $\phi_{max}$. It is important to note that for this thermal source configuration, the behavior of $\phi_{max}$ exhibits growth and not decay when the thermophoretic parameter is small; undoubtedly, natural convection does not represent a significant effect.

**Figure 9:**
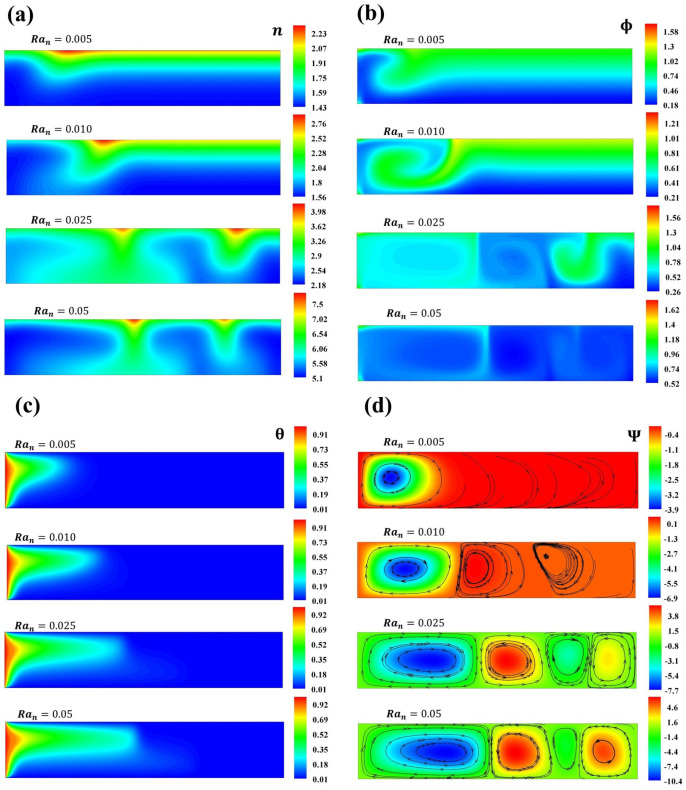
Concentration of (**a**) microorganisms and (**b**) nanoparticles, (**c**) temperature contours, and (**d**) streamlines for $Ra_{n}=0.005,0.010,0.025,0.05$.

Figure 9 shows the development of n,Ψ,Φ, and θ when $\delta_{Tm}=0.05,\delta_{Bm}=\left[0.1-0.5\right]$ as $Ra_{n}$ is increased. As mentioned above, there is no natural convection caused by a thermal source at the bottom. Therefore, at $Ra_{n}=0.01$, thermophoresis and Brownian effects disturbed the suspension, generating a plume of microorganism concentration, and recirculation of nanoparticles caused by the microorganisms swimming near the thermal source is observed. The streamlines exhibited one bioconvective cell that promoted the flow of a high particle concentration. When $Ra_{n}=0.025$, the microorganisms form two concentration plumes, the larger near the thermal source. Interestingly, there are two zones of nanoparticle concentration, one with recirculation and the other with a well-defined concentration plume and recirculation. The contour of the streamlines showed three defined rolls. For $Ra_{n}<0.1$, two concentration plumes of microorganisms are obtained. As $Ra_{n}$ increases, Brownian and thermophoretic effects impact the spatial distribution of the suspension, and two bioconvective cells are observed.

In case 1, when $\delta_{Tm}$ is constant and $\delta_{Bm}$ is varied for values above the subcritical Rayleigh $Ra_{n_{cr}}=0.03$, four rolls or cells are observed (see Figure 3 and Figure 5). While $\delta_{Bm}$ is constant and $\delta_{Tm}$ is varied, the same $Ra_{n_{c}r}=0.03$ and pattern are obtained (see Figure 3 and Figure 6). In contrast to case 2, due to thermophoretic and Brownian instability, four deformed rolls are observed when $Ra_{n_{c}r}=0.01$, as shown in Figure 8 and Figure 9.

### 4.3. From Theory to Real-World Setup

According to the results, there is better control of the swimming behavior of the microorganisms when $\delta_{Bm}=\delta_{Tm}=0.1$, when $0.005<Ra_{n}<0.015$. Here, the thermal source is at the bottom, and the small values of the thermophoretic and Brownian parameters show that the main driving force is bioconvection. However, at these values, the small concentrations of nanoparticles can travel through the streamlines that have been generated by the swimming velocity of the microorganisms, which is favorable for the removal of solids at nanometer scales where no recirculation is shown. However, the second case showed the travel of nanoparticles to the streamlines. Here, recirculations are observed which promote mixing in the aqueous suspension. In a real-world situation, this theoretical understanding could be used in a wastewater pool to enable solid removal; millions of microorganisms may be controlled with a thermal source, creating streamlines where heavy metal nanoparticles travel to an absorber or collecter of the solids. This represents a novel approach to solid removal in wastewater—saving life with life.

## 5. Conclusions

This work analyzed the numerical simulation of gravitactic bioconvection influenced by nanoparticles suspended in water. Case studies are explored involving the removal of nanometric particles suspended in water polluted by the chemical and agricultural industries. Since the swimming of *Paramecium caudatum* microorganisms in the direction of a heat source has previously been reported, this work considered the use of this source to control and orient swimming in order to remove particles that are denser than water from one zone to another. Two cases were analyzed: the former examined the relative effects of natural convection, bioconvection, and the buoyancy of nanoparticles. The latter did not consider natural convection as a result of the thermal source configuration.

The first and second cases showed the influence of heat on the swimming behavior of microorganisms, with microorganisms orienting their concentration toward heat conduction. Swimming enhances the plume concentration and nanoparticle distribution. When the microorganism plume concentrations decrease, the nanoparticles are attracted. Although natural convection does not occur, nanoparticle-perturbed bioconvection and thermal sources lead to the formation of a nanoparticle plume concentration.

At low values of the thermophoretic parameter $\delta_{Tm}<0.05$, the nanoparticles are concentrated opposite the heat source when $Ra_{n}<0.015$ and $\delta_{Bm}=0.1$. In the presence or absence of natural convection, these parameters allow us to conclude that with low concentrations of nanoparticles $Ra_{n}=\left[0.01-0.025\right]$, the microorganism *Paramecium caudatum* can be employed to remove particles with a relative density of 2.5, with a settled configuration.

The Brownian and thermophoresis effects destabilized the suspension, promoting diffusion or a higher nanoparticle concentration. The results presented provide a basis for applying two heat source configurations in a cavity. The study suggests that gravitactic microorganisms suspended in water can enable heavy metal nanoparticles to be removed. The use of a thermal source in the left corner at the bottom wall is the best option to remove nanoparticles that travel through the streamlines created by the swimming of the microorganisms, where they can be absorbed or collected.This is the first attempt to remove heavy metals nanoparticles in water suspension by microorganisms. Developing a technology of this kind represents a breakthrough in the bioremediation of contaminated water. 

## Figures and Tables

**Table 1:** Mesh independence test.

Mesh	$\phi_{\max}$, Ra=0.09	$\Psi_{\max}$, Ra=0.09	$\phi_{\max}$, Ra=0.14	$\Psi_{\max}$, Ra=0.14
$M_{1}=51\times 81$	0.52565	13.57062	0.49894	24.90609
$M_{2}=81\times 81$	0.51224	12.74131	0.46851	23.48302
$M_{3}=101\times 81$	0.51224	12.74131	0.46851	23.48302
$M_{4}=201\times 81$	0.50591	12.51656	0.45513	24.48658
$M_{5}=301\times 81$	0.50591	12.51656	0.45513	24.48658

## Data Availability

The original contributions presented in this study are included in the article. Further inquiries can be directed to the corresponding authors.

## Abbreviations

*A*	Aspect ratio	-
$D_{m}$	Diffusivity of microorganisms	m^2^s^−1^
*g*	Gravity	ms^−2^
*H*	Cavity height	m
$\tilde{J}$	Dimensionless flux of microorganisms	cellm^−2^s^−1^
*L*	Cavity width	m
Le	Lewis number	-
*n*	Microorganism concentration	cellm^−3^
$n_{b}$	Bottom concentration of microorganisms	cellm^−3^
$n_{u}$	Upper concentration of microorganisms	cellm^−3^
$\bar{n}$	Average concentration of microorganisms	cellm^−3^
$\bar{N}$	Average dimensionless concentration of microorganisms	-
Pe	Peclet number	-
Ra	Rayleigh number	-
Sc	Schmidt number	-
*t*	Dimensionless time	-
*T*	Temperature	C
$T_{c}$	Cold wall temperature	C
$T_{h}$	Hot wall temperature	C
$\tilde{u}$	Horizontal component of dimensionless velocity	ms^−1^
$\tilde{v}$	Vertical component of dimensionless velocity	ms^−1^
$V_{c}$	Upward microorganisms’ velocity	ms^−1^
$W_{o}$	Darcy velocity	ms^−1^
$x_{1},x_{2}$	Dimensionless coordinate system	-
**Greek symbols **
α	Thermal diffusivity	m^2^s^−1^
ϑ	Volume of microorganisms	m^3^
γ	Unit vertical vector	-
Ψ	Dimensionless stream function	
ϕ	Nanoparticles volume fraction	
ω	Dimensionless vorticity	
**Subscripts**		
*B*	Brownian	
*T*	Thermal	
*n*	Nanoparticles	
